# Comparative Genomics Provide Insights into Evolution of *Trichoderma* Nutrition Style

**DOI:** 10.1093/gbe/evu018

**Published:** 2014-01-29

**Authors:** Bin-Bin Xie, Qi-Long Qin, Mei Shi, Lei-Lei Chen, Yan-Li Shu, Yan Luo, Xiao-Wei Wang, Jin-Cheng Rong, Zhi-Ting Gong, Dan Li, Cai-Yun Sun, Gui-Ming Liu, Xiao-Wei Dong, Xiu-Hua Pang, Feng Huang, Weifeng Liu, Xiu-Lan Chen, Bai-Cheng Zhou, Yu-Zhong Zhang, Xiao-Yan Song

**Affiliations:** ^1^State Key Laboratory of Microbial Technology, Shandong University, Jinan, China; ^2^Marine Biotechnology Research Center, Shandong University, Jinan, China; ^3^CAS Key Laboratory of Genome Sciences and Information, Beijing Institute of Genomics, Chinese Academy of Sciences, Beijing, China; ^4^Technology Center, Shandong Tobacco Industry Corporation, Jinan, China

**Keywords:** *Trichoderma longibrachiatum*, cellulolytic enzymes, carbohydrate-active enzymes, proteases, purifying selection, d*N*/d*S*

## Abstract

Saprotrophy on plant biomass is a recently developed nutrition strategy for *Trichoderma*. However, the physiology and evolution of this new nutrition strategy is still elusive. We report the deep sequencing and analysis of the genome of *Trichoderma longibrachiatum*, an efficient cellulase producer. The 31.7-Mb genome, smallest among the sequenced *Trichoderma* species, encodes fewer nutrition-related genes than saprotrophic *T. reesei* (*Tr*), including glycoside hydrolases and nonribosomal peptide synthetase–polyketide synthase. Homology and phylogenetic analyses suggest that a large number of nutrition-related genes, including GH18 chitinases, β-1,3/1,6-glucanases, cellulolytic enzymes, and hemicellulolytic enzymes, were lost in the common ancestor of *T. longibrachiatum* (*Tl*) and *Tr*. d*N*/d*S* (ω) calculation indicates that all the nutrition-related genes analyzed are under purifying selection. Cellulolytic enzymes, the key enzymes for saprotrophy on plant biomass, are under stronger purifying selection pressure in *Tl* and *Tr* than in mycoparasitic species, suggesting that development of the nutrition strategy of saprotrophy on plant biomass has increased the selection pressure. In addition, aspartic proteases, serine proteases, and metalloproteases are subject to stronger purifying selection pressure in *Tl* and *Tr*, suggesting that these enzymes may also play important roles in the nutrition. This study provides insights into the physiology and evolution of the nutrition strategy of *Trichoderma*.

## Introduction

*Trichoderma* (telemorph *Hypocrea*) species are highly interactive in root, soil, and foliar environments and are among the most commonly isolated saprotrophic fungi (Harman et al. 2004; Druzhinina et al. 2011). Most *Trichoderma* can grow on both living fungi (mycoparasitism) and dead fungal substances (saprotrophy on fungal substances) and their nutrition strategy is referred to as mycotrophy (Druzhinina et al. 2011). Because of many preys are plant pathogenic fungi, some *Trichoderma* species, for example, *Trichoderma atroviride* (*Ta*) and *T. virens* (*Tv*), are used as biocontrol reagents (Harman et al. 2004). Though mycotrophy is considered as the ancestral and the major lifestyle for *Trichoderma* (Kubicek et al. 2011), it is also noted that, several recent taxa of the genus, which occupy terminal positions in the phylogenetic trees, seem to have shifted to new ecological niches (Druzhinina et al. 2011). For example, *T. reesei* (*Tr*) specializes on colonizing dead wood, *T. longibrachiatum* (*Tl*) can colonize immunocompromised humans, and some species are isolated as endophytes (symptomless growth inside plant tissue) (Druzhinina et al. 2011).

Comparative genomics of *Ta*, *Tv*, and *Tr* suggests that, mycoparasitic species have a large set of mycoparasitism-related genes, including carbohydrate-active enzymes (CAZymes) and secondary metabolism-related genes (Kubicek et al. 2011). In comparison, saprotrophic species *Tr* has a smaller set of CAZymes and secondary metabolism-related genes, consistent with its lower mycoparasitic ability. Phylogenetic analysis suggests that the mycotroph-related genes arose in the common ancestor of *Trichoderma,* which had the ancestral life style of mycotrophy, and some of these genes were subsequently lost in saprotrophic *Tr* (Kubicek et al. 2011). This conclusion is consistent with the hypothesis that *Tr* became an efficient saprotroph on dead wood by following wood-degrading fungi into their habitat (Rossman et al. 1999). Currently, because the genome sequences for other *Trichoderma* species, especially those closely related to *Tr*, are unavailable, it is still unclear whether similar genome reduction occurs for other *Trichoderma* species.

*Trichoderma* sp. SMF2, firstly published as *T. koningii*, is a biocontrol fungus that has a strong inhibitory ability against plant pathogenic fungi and Gram-positive bacteria (Song et al. 2006). It was reclassified as *T. pseudokoningii* based on molecular data and morphological data (Chen et al. 2009). The secondary metabolites, peptaibols, and the extracellular enzymes secreted by SMF2 are thought to be important factors that contribute to the inhibitory ability against pathogens. Peptaibols (Song et al. 2007; Luo et al. 2010; Shi et al. 2010; Su et al. 2012) and proteases (Chen et al. 2009) secreted by SMF2 are used to study the biocontrol mechanism of *Trichoderma* in our laboratory. To gain insights into the physiology and the biocontrol mechanism, the genome of SMF2 was deeply sequenced and the nutrition-related genes including those of chitinases, β-1,3/1,6-glucanases, cellulolytic enzymes, hemicellulolytic enzymes, and proteases were systematically annotated. Furthermore, SMF2 was reclassified as *Tl* based on phylogenetic analysis with *tef1*, *cal1*, and *chi18-5* genes as molecular markers.

Both *Tl* and *Tr* belong to the Longibrachiatum clade of *Trichoderma* (Druzhinina et al. 2012; Samuels et al. 2012). Members of this clade are best known as producers of cellulose hydrolyzing enzymes (for *Tr* and *Tl*), as cause of opportunistic infections of man and animals (for *Tl* and close relatives), and for their association with wet building materials (Samuels et al. 2012). To gain further insights into the genome evolution and the genetic basis underlying the evolution of nutrition strategy, *Tl* genome was compared with that of *Tr*, *Ta*, and *Tv*. Our results show that, nutrition strategy is not only related to the number of nutrition-related genes but also affect the selection pressure on these genes. This study will improve our understanding of the physiology and evolution of *Trichoderma* nutrition style.

## Materials and Methods

### Fungal Strains and Cultivation Conditions

For genome sequencing, *Tl* SMF2 was grown in 0.2% potato dextrose medium (Sigma, USA), with shaking at 120 rotations per minute for 72 h at 28 °C.

### Genome Sequencing and Assembly

For genome sequencing, fungal mycelia were collected at 72 h from 0.2% potato dextrose medium. DNA was prepared using E.Z.N.A. Fungal DNA Mini Kit (OMEGA, USA) and was freeze-dried for genome sequencing. Using a combination of Roche 454 and Illumina Solexa technologies, *Tl* genome was sequenced to an average of 69-fold coverage. A shotgun library was sequenced with Roche 454, resulting 2,555,045 reads (852,363,657 bp). Two paired-end libraries (200 bp and 2 kb) were sequenced using Illumina Solexa (read length, 44 bp), producing 15,719,200 clean reads (691,644,800 bp, 200 bp library) and 14,538,236 clean reads (639,682,384 bp, 2 kb library), respectively. The 454 reads and Solexa reads were assembled together using MIRA v3.4.0 (Chevreux et al. 1999), which resulted in an assembly of 31,735,570 bp in 365 large contigs (≥1 kb). These large MIRA contigs were then assembled into the final assembly with the help of the paired-end information using SSPACE basic v2.0 (Boetzer et al. 2011).

Assembly sequences, gene coordinates, and annotation data for *Tl* are available through anonymous ftp (ftp://222.206.24.193, last accessed February 11, 2014). The genome assembly has been deposited at DDBJ/EMBL/GenBank under the accession (GenBank: ANBJ00000000). The version described in this article is the first version (GenBank: ANBJ01000000).

### Genome Data for Comparative Analysis

*Trichoderma* genome sequence files and gene coordinate files were downloaded from Department of Energy Joint Genome Institute (JGI) Genome Portal (http://genome.jgi.doe.gov/, last accessed February 11, 2014) for *Tr* (http://genome.jgi.doe.gov/Trire2/Trire2.home.html, last accessed February 11, 2014), *Tv* (http://genome.jgi.doe.gov/TriviGv29_8_2/TriviGv29_8_2.home.html, last accessed February 11, 2014), and *Ta* (http://genome.jgi.doe.gov/Triat2/Triat2.home.html, last accessed February 11, 2014). Annotations were downloaded from GenBank (*Tr*, AAIL02000000; *Tv*, ABDF02000000; and *Ta*, ABDG02000000). *Fusarium graminearum* data were downloaded from *Fusarium* Comparative Sequencing Project, Broad Institute of Harvard and Massachusetts Institute of Technology (http://www.broadinstitute.org/, last accessed February 11, 2014). *Neurospora crassa* data were downloaded from *N. crassa* Sequencing Project, Broad Institute of Harvard and Massachusetts Institute of Technology (http://www.broadinstitute.org/, last accessed February 11, 2014).

### Gene Prediction

First, models were predicted using the de novo predictor Fgenesh, version 2.6 (Salamov and Solovyev 2000) with parameters trained for fungi *Fusarium*/Pezizomycotina. Then, regions without Fgenesh models were searched against the gene sets of the three published *Trichoderma* genomes, with BlastX. The top BlastX hits (identity above 50%) were used to predicted gene models with the help of Genewise (Birney and Durbin 2000). Genes shorter than 100 bp were excluded. As a result, we obtained 9,409 nonredundant models.

### Gene Annotation

Protein sequences were searched against SwissProt database (http://www.ebi.ac.uk/uniprot/, last accessed February 11, 2014) using BlastP, with *E* value ≤ 1*E*−10, alignment identity ≥ 35%, and alignment score ≥ 60 as filters. In addition, length of alignment must be longer than half of the query length and target length, and the difference of the query and target length must be shorter than 25% of the shorter one. As a result, 2,981 proteins have a match in SwissProt. The metabolic pathways were annotated using KEGG (Kanehisa et al. 2004) on KAAS server (http://www.genome.jp/tools/kaas/, last accessed February 11, 2014), with Bidirectional-Best-Hit method and fungal genomes as reference. The protein domains were annotated using Pfam database version 26.0 and program pfamscan. NRPS and PKS genes were identified using SMURF server (http://www.jcvi.org/smurf/index.php, last accessed February 11, 2014) (Khaldi et al. 2010).

The tRNA genes were predicted using tRNAscan-SE, version 1.3 (Schattner et al. 2005). The rRNA genes were predicted using RNAmmer 1.2 Server (http://www.cbs.dtu.dk/services/RNAmmer/, last accessed February 11, 2014) (Lagesen et al. 2007).

### Reclassification of SMF2 Based on Molecular Phylogeny

Two methods have been used to reclassify SMF2. Firstly, SMF2 was reclassified using the server TrichOKEY v. 2.0 (http://isth.info/, last accessed February 11, 2014) (Druzhinina et al. 2005), which uses a combination of several oligonucleotides allocated within the internal transcribed spacer 1 and 2 (ITS1 and 2) sequences of the rDNA repeat to quickly identify *Hypocrea*/*Trichoderma* at the genus and species levels. With the ITS sequence of SMF2 (GenBank Accession FJ605099.1) as input, SMF2 was classified as *Tl* or *Hypocrea orientalis*, both with high identification reliability as reported by the server.

Then, we classify SMF2 using the *tef1*, *cal1*, and *chi18-5* genes (Druzhinina et al. 2012). The DNA sequences for the above genes for the species in the section Longibrachiatum of *Trichoderma* were obtained from National Center for Biotechnology Information (NCBI) GenBank based on the accessions listed in the study of Druzhinina et al. (2012). The corresponding gene sequences in SMF2 genome were obtained by searching against the predicted gene sequences of SMF2 using BlastN. Then, the sequences for each gene were aligned with Muscle version 3.8.31 (Edgar 2004), separately. Each alignment was visually checked with the help of BioEdit (Hall 1999). To obtain a better phylogenetic tree, several short sequences were excluded from the alignment. Thirdly, the poorly aligned regions were removed from each alignment using Gblocks version 0.91 b (Castresana 2000) with default parameters for nucleotide sequences. The sequences for each strain were checked and only strains for which the *tef1*, *chi18-5,* and *cal1* genes were all included in the alignments were used to create a concatenated alignment of the three genes. Finally, the concatenated alignment (including 952 sites) for the three genes was used to construct phylogenetic tree using Metropolis-coupled Markov chain Monte Carlo sampling with MrBayes version 3.2.2 (Ronquist et al. 2012). The GTR + I + Γ nucleotide substitution model was used, and two simultaneous runs of four incrementally heated chains were performed for 5 millions of generations. The accessions for the sequences used in this study were listed in supplementary table S17, Supplementary Material online.

### Orthologous Genes

The orthologous gene families in the four *Trichoderma* species were analyzed using BlastP and OrthoMCL (Li et al. 2003), with *E*-value cutoff 1*E*−5, identity cutoff 50% and index *I* = 1.5.

To construct a phylogenetic tree for the *Trichoderma* spp. based on the protein sequences, we first computed the orthologous gene groups for four *Trichoderma* spp. and *F. graminearum* genome and *N. crassa* genome using OrthoMCL (Li et al. 2003), with *E*-value cutoff 1*E*−5, percentage identity cutoff 50% and index *I* = 1.5. As a result, 5,145 homologous groups with each genome having one orthologous gene were obtained. Then, each group was aligned separately using Muscle version 3.8.31 (Edgar 2004), and the aligned sequences of all the groups were concatenated and conserved blocks were obtained for phylogenetic analysis using Gblocks version 0.91 b (Castresana 2000) with default parameters. The final alignment contained 2,143,124 sites and was used to construct a neighbor-joining tree using MEGA version 5.05 (Tamura et al. 2011), with JTT model and 500 sets of bootstrap replications.

### Annotation of CAZymes

We firstly retrieved CAZyme sequences based on GenBank annotations of *Tr*, *Ta*, and *Tv* genomes and annotated Pfam domains in these sequences. Then, the discovered Pfam domains were checked against CAZy and those unambiguously affiliated to a CAZy family were used to compile a dictionary (supplementary table S6, Supplementary Material online) to further identify CAZymes from the four *Trichoderma* genomes. Several additional Pfam domains were also manually incorporated into the dictionary to predict more CAZymes. Finally, the four *Trichoderma* genomes were annotated using Pfam and the sequences with a Pfam domain in the dictionary were classified as CAZy.

### Evolutionary Changes of Numbers of Nutrition-Related Genes

**Fig. 1.—:**
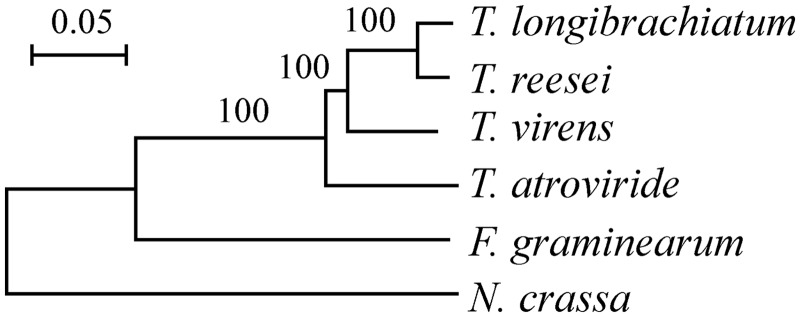
A consensus neighbor-joining tree for *Tl* and close relatives. The tree was created based on 2,143,124 sites in 5,145 orthologous proteins using JTT matrix and 500 bootstrap replications. Bootstrap percentages were shown on the branches. The bar represents 0.05 substitutions per site.

**Fig. 2.—:**
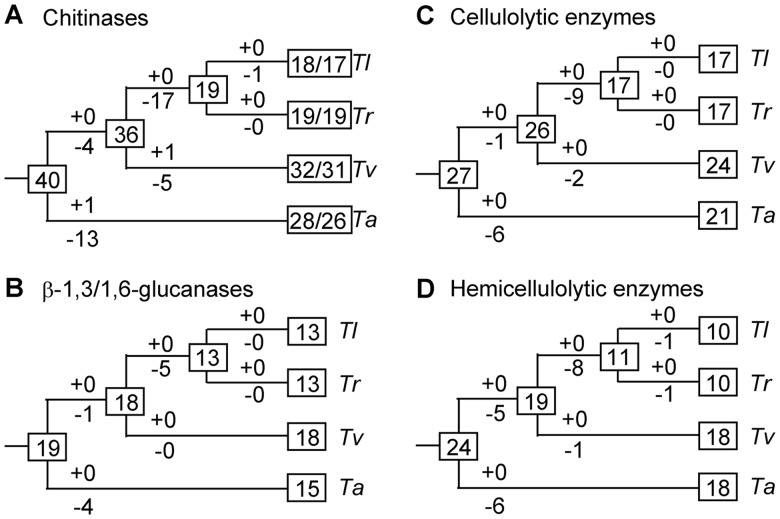
Gains and losses of genes for chitinases (*A*), β-1,3/1,6-glucanases (*B*), cellulolytic enzymes (*C*), and hemicellulolytic enzymes (*D*). Numbers in boxes indicate the numbers of genes in the extent and ancestral species. Numbers with plus and minus signs on branches indicate gene gains and losses. For chitinases (*A*), gene gains and losses were estimated based on the sequences of chitinases and the closely related homologs (see Materials and Methods). Two gene numbers were presented for the extent species, with the left one indicating the numbers of chitinases plus the closely related homologs and the right one indicating the numbers of chitinases.

Gene numbers of chitinases, β-1,3/1,6-glucanases, cellulolytic enzymes, and hemicellulolytic enzymes in the ancestral species and gains and losses of these genes along each lineage were estimated using the reconciled tree method (Goodman et al. 1979; Page and Charleston 1997; Nam and Nei 2005; Niimura and Nei 2007). This method finds out the differences between a species tree and the phylogenetic tree of a gene family and then fits the gene tree into the species tree by modeling these differences as gene gains and losses parsimoniously (i.e., to find out the minimum number of gene duplications plus gene losses). For each type of enzymes, the sequences were firstly grouped based on the homologous gene families calculated using BlastP and OrthoMCL (Li et al. 2003). Sequences of each group were aligned using Muscle version 3.8.31 (Edgar 2004) and used to construct a neighbor-joining tree using MEGA version 5.05 with JTT model and 500 bootstrap replicates. The groups containing too distantly related sequences were further divided into subgroups by checking the length and bootstrap support for each branch of the neighbor-joining tree. The neighbor-joining tree was processed by the c program branchout, and the output file was then used to count the gene changes using the perl script mrcacount.pl (Niimura and Nei 2007). A bootstrap cutoff of 70% was used in the analysis. The phylogenetic tree in figure 1 was used as the species tree. Both program branchout and perl script mrcacount.pl were kindly provided by Yoshihito Niimura. The number of genes in each ancestral node and the gene gain and loss events along each branch were summarized over all the groups and subgroups to get the results present in figure 2. For chitinases, the BlastP and OrthoMCL analyses revealed four closely related homologs (one for *Tr*, two for *Ta*, and one for *Tv*). These homologs do not contain a Pfam domain that was used to classify CAZymes, and therefore, they were not included in tables 1 and 2. In spite of this annotation inconsistence, these homologs were used in estimation of gene gains and losses in chitinase evolution.

**Table 1:** Comparison of CAZymes of *Trichoderma*

Species	GH	GT	PL	CE	Total
*Tl*	165	93	4	19	281
*Tr*	174	94	4	19	291
*Ta*	218	97	8	26	349
*Tv*	233	101	7	26	367

**Table 2:** Comparison of Major Nutrition-Related CAZymes of *Trichoderma*

Species	Fungal Cell Wall Polysaccharides-Degrading Enzymes		Plant Cell Wall Polysaccharides-Degrading Enzymes	
	Chitinases	β-1,3/1,6-Glucanases	Cellulolytic Enzymes	Hemicellulolytic Enzymes
*Tl*	17	13	17	10
*Tr*	19	13	17	10
*Ta*	26	15	21	18
*Tv*	31	18	24	18

### Annotation of Proteases

Proteases were annotated by searching against the peptidase database MEROPS Release 9.6 (http://merops.sanger.ac.uk/, last accessed February 11, 2014) (Rawlings et al. 2012) using BlastP, with identity cutoff 35%, *E*-value cutoff 1*E*−5, and score cutoff 30. Sequences whose best hit was a “nonpeptidase homolog” or “peptidase inhibitor” were discarded.

### Calculation of d*N* and d*S*

For the calculation of d*N* and d*S*, each homology group of protein sequences were aligned using Muscle version 3.8.31 (Edgar 2004). Gap-containing columns were removed from the amino acid alignment. Then the nucleotide sequence alignment for each homology group was created by getting the corresponding codon from the gene sequence for each residue in the amino acid alignment. Pair-wise nucleotide alignments were obtained by directly retrieving the sequences in the alignment of the homology group. d*N*, d*S*, and d*N*/d*S* (ω) values were calculated using KaKs_Calculator with MS model (Zhang et al. 2006). The homology groups with d*N* or d*S* values >2 were excluded from the data since these too high substitution rates are probably poorly estimated. Those with *P* value > 0.001 were also excluded to get reliable results.

## Results

### Sequencing of SMF2 Genome

SMF2 genome was sequenced to an average of 69-fold coverage using a combination of Roche 454 and Illumina Solexa technologies (supplementary table S1, Supplementary Material online). The final assembly contains 316 contigs in 185 scaffolds with a total size of 31,747,380 bp (including 13,491 *N*’s, see supplementary table S2, Supplementary Material online, for details of current assembly of SMF2 genome). The 31.7-Mb genome of SMF2 is the smallest among the four sequenced *Trichoderma* spp. (table 3) and similar to that of *Tr* (33.9 Mb). GC content of the assembly is 54.0%, the highest among the sequenced *Trichoderma* spp. (52.7% for *Tr*, 49.7% for *Ta,* and 49.2% for *Tv*).

**Table 3:** Comparison of *Trichoderma* Genomes

Species	Size^a^ (Mb)	Coverage^a^	Gaps^a^ (Mb)	Scaf. Num.^a^	GC%^b^	Gene Num.^a^	Gene Len.^a^ (bp)
*Tl*	31.7	69×	0.01	185	54.0	9,409	1,654
*Tr*	33.9	9.0×	0.05	89	52.8	9,143	1,793
*Ta*	36.1	8.3×	0.1	50	49.7	11,865	1,747
*Tv*	39.0	8.1×	0.2	135	49.2	12,518	1,710

^a^Data for *Tr*, *Ta*, and *Tv* were adopted from Kubicek et al. (2011).

^b^Data for *Tr*, *Ta*, and *Tv* were calculated based on sequence data obtained from DOE JGI (http://genome.jgi-psf.org/, last accessed February 11, 2014).

A combination of an ab initio gene predictor (e.g., Fgenesh [Salamov and Solovyev 2000]) and a homology-based gene predictor (e.g., Genewise (Birney and Durbin 2000) and Fgenesh+ [http://www.softberry.com, last accessed February 11, 2014]) was used to predict protein-coding genes. The final gene set comprised 9,409 models, including 5,779 complete models (with both start codon and stop codon) plus 3,630 partial models (without start codon and/or stop codon). This number is slightly larger than that of *Tr* (9,129), but much smaller than that of *Ta* (11,863) and *Tv* (12,427). This is consistent with the similar size of SMF2 and *Tr* genomes.

A full list of annotation information was included in supplementary table S3, Supplementary Material online.

### Reclassification of SMF2 as T*l*

**Fig. 3.—:**
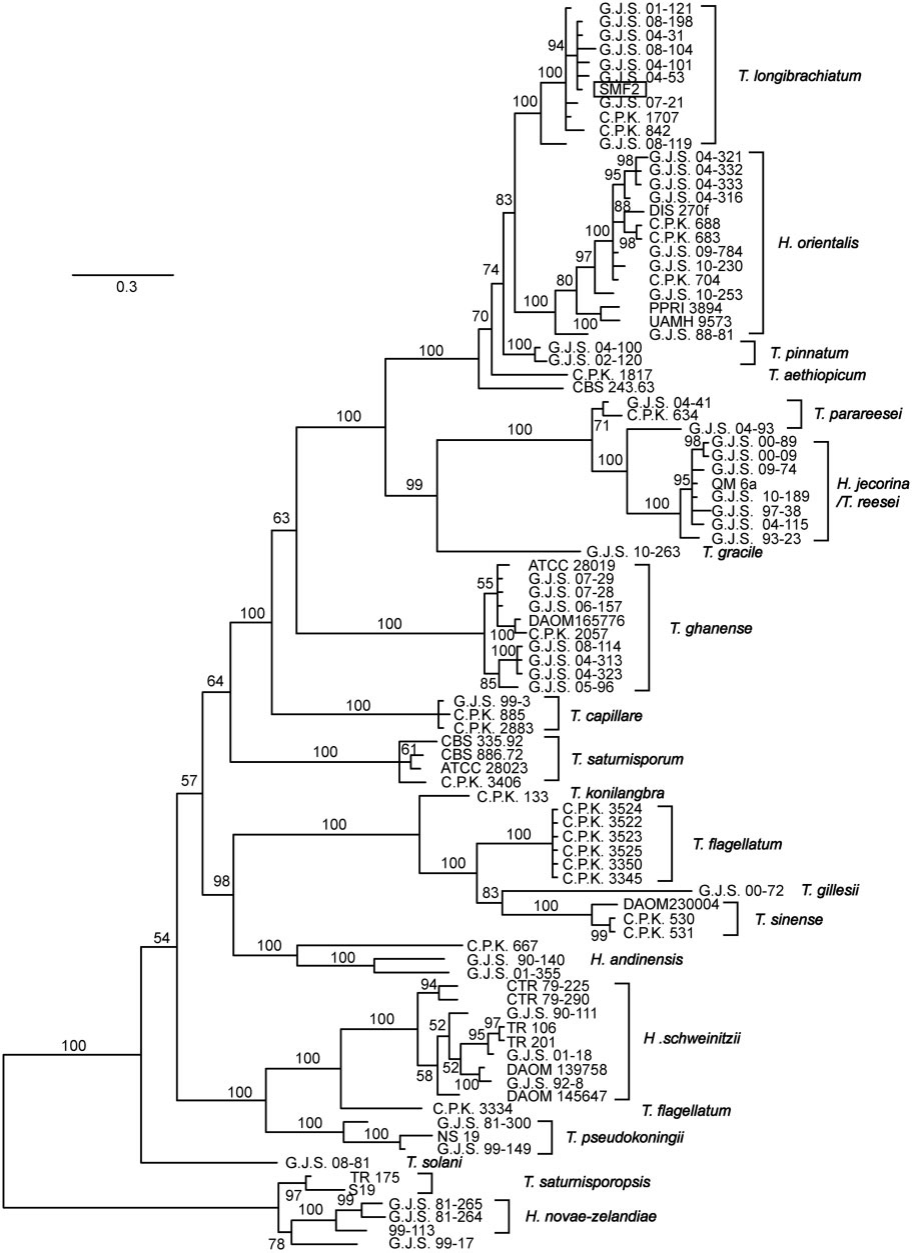
Bayesian phylogenetic tree based on the concatenated alignments of *tef1*, *cal1,* and *chi18-5* genes. Posterior probability (>0.5) are shown as percentages.

In this study, SMF2 was reclassified based on molecular data. Firstly, SMF2 was classified using the server TrichOKEY v. 2.0 (http://isth.info/, last accessed February 11, 2014) (Druzhinina et al. 2005). With the internal transcribed spacer sequence (GenBank Accession FJ605099.1) as input, SMF2 was classified as “*Trichoderma longibrachiatum*–*Hypocrea orientalis.*” To further clarify the taxonomy of SMF2, the concatenated sequence of *tef1* (SMF2FGGW_107796), *cal1* (SMF2FGGW_102813), and *chi18-5* (SMF2FGGW_107557) genes were used to construct a Bayesian phylogenetic tree as described (Druzhinina et al. 2012). As shown in figure 3, the Bayesian phylogenetic tree included SMF2 and another 91 strains, representing 20 formally described species plus four phylogenetic species and lone lineages within Longibrachiatum clade. All the species except *T. parareesei* and *T. flagellatum* are monophyletic, with the branches being supported by posterior probability >0.5. SMF2 is clustered within the branch of *Tl* with posterior probability of 1. Taken the above results together, strain SMF2 is reclassified as *Tl*.

### Protein Families Expanded and Shrunken in T*l*

Based on Pfam domain annotation, the protein families were compared for the four *Trichoderma* species (supplementary table S4, Supplementary Material online). A protein number difference of ≥2 or ≤−2 was used as the criteria of expansion or shrunk. *Tl* was compared with the other three *Trichoderma* species *Tr*, *Ta*, and *Tv*, which revealed that 42, 22, and 9 families were expanded and 44, 147, and 325 families were shrunken. Recently developed species *Tl* and *Tr* were also compared with the early branched species *Ta* and *Tv*. Results showed that, compared with *Ta* and *Tv*, 175 families were shrunken in *Tl* and *Tr,* and no families were expanded (supplementary table S4, Supplementary Material online). The most shrunken families include proteins containing zinc finger (Zn_clus, PF00172), proteins containing ankyrin repeats (Ank_2, PF12796, Ank, PF00023), fungal-specific transcription factors (Fungal_trans, PF04082, Fungal_trans_2, PF11951), the major facilitator superfamily transporters (MFS_1, PF07690), sugar (and other) transporter (Sugar_tr, PF00083), proteins containing NTPase domain (NACHT, PF05729), short chain dehydrogenases (adh_short, PF00106), proteins containing alcohol dehydrogenase GroES-like domain (ADH_N, PF08240), zinc-binding dehydrogenases (ADH_zinc_N, PF00107), alpha/beta hydrolases (Abhydrolase_6, PF12697), subtilases (Peptidase_S8, PF00082), NmrA-like negative transcriptional regulators (NmrA, PF05368), heterokaryon incompatibility proteins (HET, PF06985), phosphorylase superfamily proteins (PNP_UDP_1, PF01048), and proteins containing WD40 repeats (WD40, PF00400).

### Orthologous Genes in the Four Sequenced *Trichoderma* Genomes

We used BlastP and OrthoMCL (Li et al. 2003) to analyze the orthologous genes among the four sequenced *Trichoderma* spp. Because the genome sequences of the four species are all incomplete, the gene numbers and the homology family numbers are underestimated. However, the high percentage of core gene families suggests that most of the genes are included in the current assembly of genome sequence.

**Fig. 4.—:**
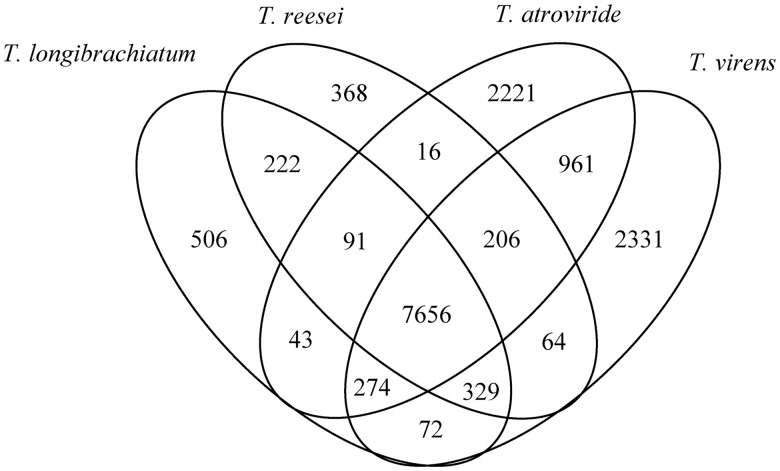
Venn diagrams of homologous genes in four *Trichoderma* species. Numbers in circles indicate the numbers of homologous protein families.

Results showed that all the 42,828 genes from the four genomes are classified into 15,360 families (fig. 4; see supplementary table S5, Supplementary Material online, for a full list of the homology families). The four species have a large core gene set of 7,656 families. The core family number can be extended to 8,556 if the families absent from one species were considered. Out of the 7,656 core families, 7,411 (96.8%) have equal number of homologous genes in each species (including 7,361 “1:1:1:1”-type, 44 “2:2:2:2”-type, four “3:3:3:3”-type, and two “4:4:4:4”-type families). There were 506 *Tl*-specific families, most of which were not annotated by searching against swiss-prot and KEGG. The annotated proteins include a zinc-type alcohol dehydrogenase-like protein, a family GH89 glycoside hydrolase (GH), and a number of peptidases, including a family M4 metallopeptidases, a family S9 serine peptidase, a peptidase Clp {type 1}, a family M20A metallopeptidase, a family C26 cysteine peptidase, and a family S12 serine peptidase. It has been suggested that nitrate reductase may be helpful for *Trichoderma* species survive on nitrogen-derived decaying wood (Slot and Hibbett 2007). Annotation revealed a nitrate reductase (SMF2FGGW_103702) and a molybdopterin molybdotransferase (SMF2FGGW_103701) in the *Tl*-specific families. Genes encoding these two enzymes form a cluster in the gene genome, suggesting that this nitrate reductase may be functional. In addition to this nitrate reductase, *Tl* genome encodes another two nitrate reductases. One nitrate reductase (SMF2FGGW_107092), which forms a cluster with a nitrite reductase (SMF2FGGW_107093) and a nitrate/nitrite transporter (SMF2FGGW_107094), is present in all the four *Trichoderma* species, and the other (SMF2FGGW_107094) is present in *Ta* and *Tv* but not in *Tr*. The presence of these nitrate reductases suggests a high acquiring ability of *Tl*.

Pair wise comparison showed that, mycoparasitic species *Ta* and *Tv* share the largest number of groups (9,097). *Tl* shares more families with *Tv* (8,316) and *Tr* (8,262), than with *Ta* (8,043), suggesting *Tl* has a closer relationship with *Tv* and *Tr* than with *Ta*. To clarify the phylogenetic position of *Tl*, a neighbor-joining tree was constructed based on the 5,145 orthologous proteins (alignment including 2,143,124 sites without gaps) present in all the four *Trichoderma* spp. and fungi *Fusarium graminearum* and *Neurospora crassa* genomes (fig. 1). It was shown that *Tl* has the closest relationship to *Tr* among the studied genomes. Considering *Tl* and *Tr* share a large ratio of genes, it is likely that the loss of mycoparasitism-related genes may occur before the speciation of *Tl* and *Tr*.

### Carbohydrate-Active Enzymes

CAZymes play key roles in the degradation of plant cell wall polysaccharides and fungal cell wall polysaccharides by *Trichoderma* spp. (Martinez et al. 2008; Kubicek et al. 2011). Here, we carefully annotated the CAZymes in *Tl* as well as in *Tr*, *Ta*, and *Tv* using Pfam database (see Materials and Methods for detail). The Pfam domains used in the annotation and classification were listed in supplementary table S6, Supplementary Material online. Summary of annotation results was presented in table 1. Details for CAZy families were shown in supplementary tables S7–S11, Supplementary Material online.

Though the numbers of annotated CAZymes for *Tr*, *Tv*, and *Tv* are different from that in previous study (Kubicek et al. 2011) (supplementary table S7, Supplementary Material online), the same conclusion can be drawn that *Tr* has less GHs, glycosyltransferases (GT), polysaccharide lyases (PL), and carbohydrate esterases (CE) than *Ta* and *Tv*. Annotation of *Tl* genome revealed that, compared with *Tr*, *Tl* has a smaller number of GH (165 vs. 174), a similar number of GT (94 vs. 95), and the same numbers of PL and CE. Therefore, *Tl* has the smallest set of CAZymes among the studied *Trichoderma* species.

One key process in mycoparasitism is the lysis of the prey’s cell walls (Howell 2003; Harman et al. 2004; Lorito et al. 2010). Fungal cell wall is mostly composed of chitin, and therefore, chitinolytic enzymes are a key factor in the mycoparasitic attack (Harman et al. 2004; Seidl 2008). Previous study has shown that, consistent with weak mycoparasitic ability, saprotrophic species *Tr* has less GH18 chitinases than mycoparasitic species (Kubicek et al. 2011). Pfam annotation came to the same conclusion (table 2; supplementary tables S12–S14, Supplementary Material online, for details of the nutrition-related CAZymes). In addition, annotation of *Tl* genome showed that, *Tl* encodes less GH18 chitinases than *Tr* (17 vs. 19). See supplementary figure S1, Supplementary Material online, for a phylogenetic tree of the GH18 chitinases. Previous study has shown that *Tr* has fewer GH75 chitosanases than mycoparasitic species (Kubicek et al. 2011). Annotation of *Tl* genome revealed the same number of chitosanase genes (8) as in *Tr*. In addition to chitin, the central core of the cell wall of almost all fungi contains β-1,3/1,6-glucan (Latge 2007). Therefore, β-1,3-glucanases and β-1,6-glucanases were also annotated. Results showed that, *Tl* and *Tr* genome encode the same numbers of β-1,3-glucanases and β-1,6-glucanases (13 in all; table 2; supplementary table S12, Supplementary Material online).

The ability of saprotrophy on plant cell wall polysaccharides depends on the production of cellulolytic enzymes and hemicellulolytic enzymes. Annotation revealed that *Tl* and *Tr* genomes encode the same numbers of cellulolytic enzymes and hemicellulolytic enzymes (17 and 10; table 2; supplementary tables S13 and S14, Supplementary Material online). It was also noted that, *Tl* and *Tr* genomes encode fewer cellulolytic and hemicellulolytic enzymes than *Tv* and *Ta*.

We further estimated the gene duplication and loss events for the chitinases, β-1,3/1,6-glucanases, cellulolytic enzymes, and hemicellulolytic enzymes during the speciation of *Trichoderma* using a parsimony method (see Materials and Methods). We created phylogenetic trees for all groups/subgroups of chitinases (26 groups plus 6 subgroups), β-1,3/1,6-glucanases (16 groups plus 5 subgroups), cellulolytic enzymes (25 groups), and hemicellulolytic enzymes (17 groups plus 6 subgroups). Phylogenetic tree in figure 1 was used as a species tree. By finding out the difference between the gene tree and the species tree and fitting the gene tree into the species tree, the number of genes in each ancestral node and the gene duplications and losses along each branch of the species tree were counted.

As shown in figure 2A–D, there are 40 chitinase genes, 19 β-1,3/1,6-glucanase genes, 27 cellulolytic enzyme genes, and 24 hemicellulolytic enzyme genes in the most recent common ancestor (MRCA) of the four *Trichoderma* species, all of which are more than those in the current species. Gene loss events dominate the gene number fluctuation of all the four classes of enzymes during the evolution of the four species of *Trichoderma*. Only two chitinase genes (one in *Ta* and the other in *Tv*) were duplicated in the evolution of the four classes of nutrition-related enzymes. Therefore, for each class of enzymes, the total number of lost genes in the evolution can be estimated from the number of genes in the extent species (i.e., by subtracting the number of genes in the extent species from the number of genes in the MRCA of four *Trichoderma* species). For both fungal cell wall polysaccharides-degrading enzymes and plant cell wall polysaccharides-degrading enzymes, the most significant gene losses occurred in the MRCA of the *Tl* and *Tr*, where 26.3–42.5% of the total number of genes in the MRCA of four *Trichoderma* species were lost.

### Secondary Metabolism

Secondary metabolites are probably related to the mycoparasitism of *Trichoderma* spp. Genes related to secondary metabolism of *Tl* were predicted using SMURF (Khaldi et al. 2010). For the convenience of comparison, *Tr*, *Ta*, and *Tv* genomes were also analyzed using SMURF. As shown in table 4, *Tl* genome encodes the smallest number (22) of nonribosomal peptide synthetase (NRPS)–polyketide synthase (PKS) genes among the four sequenced *Trichoderma* genomes (27 for *Tr*, 41 for *Ta*, and 57 for *Tv*). The fewer number of NRPS/PKS genes in *Tl* than *Tr* suggests that secondary metabolism of *Tl* is probably simpler than that of *Tr*. In addition, annotation using Pfam databases indicated that the domain structure of NRPS is also different for different species, suggesting that there are large differences between the secondary metabolites of different *Trichoderma* (see supplementary table S3, Supplementary Material online, for details of Pfam annotation).

**Table 4:** Comparison of NRPS, PKS, and NRPS/PKS of *Trichoderma*

Species	NRPS	PKS	NRPS/PKS	NRPS-Like	PKS-Like	Total
*Tl*	6	9	2	4	1	22
*Tr*	8	11	2	5	1	27
*Ta*	12	18	2	8	1	41
*Tv*	19	20	2	14	1	56

The longest gene in *Tl* genome, SMF2FGGW_105489 (69,506 bp), was predicted to be a hybrid NRPS/PKS gene by SMURF. Like long NRPS genes in other *Trichoderma* spp. (Wiest et al. 2002), it is an intron-less gene, which contains only two introns. Pfam analysis suggested that the protein (23,045 aa) encoded by SMF2FGGW_105489 is responsible for synthesis of 20-aa peptaibols. The second longest gene in *Tl* genome is an NRPS gene of 43,447-bp long (SMF2FGGW_101095). It is also an intron-less gene, which contains only two introns. Pfam analysis suggested that it encodes an NRPS for the synthesis of 12-aa peptaibols. The above results agree well with our previous studies that show that *Tl* can produce a large amount of 20-aa peptaibols and a small amount of 12-aa peptaibols (Song et al. 2006, 2007). Compared with *Tl*, the longest NRPS encoded by the other three sequenced *Trichoderma* genomes are shorter, and accordingly, lengths of the longest peptaibols synthesized by the other three *Trichoderma* spp. are shorter (18 aa for *Tr* and *Tv* and 19 aa for *Ta*).

### Proteases

Proteases are important enzymes that may be related to cell wall degradation for both pathogenic fungi and pathogenic animals. Recent comparative transcriptomics of *Tr*, *Tv*, and *Ta* showed that expression of proteases is up-regulated during confrontations with a plant pathogenic fungus *Rhizoctonia solani*, indicating that proteases may play roles in the antagonism against pathogenic fungi (Atanasova et al. 2013). However, proteases of *Trichoderma* have not been systematically compared at the whole-genome scale. Here, we systematically annotated protease genes in *Tl*, *Tv*, *Tv*, and *Ta* genomes using MEROPS database (Rawlings et al. 2012). The results (table 5) showed that, *Tl* (238) and *Tr* (239) have a smaller set of proteases than *Tv* (318) and *Ta* (335). Serine proteases contribute to the majority (∼80%) of the total difference, whereas metalloproteases contribute to over 10% of the total difference. Based on the MEROPS classification, S08, S09, and S33 are the largest families of annotated serine proteases (see supplementary table S15, Supplementary Material online, for complete lists of proteases of different families). S08 family proteases, also known as subtilisin-like proteases, were found to play roles in the mycoparasitism of *Trichoderma* (Atanasova et al. 2013). *Tv* and *Ta* have more S08 proteases than *Tl* and *Tr*. In addition, *Tv* and *Ta* also have more S09, S12, S33, and S53 proteases, suggesting that these families may also contribute to the mycoparasitism of *Trichoderma*.

**Table 5:** Comparison of Proteases of *Trichoderma*

Species	A	C	G	M	S	T	Total
*Tl*	15	36	4	64	98	21	238
*Tr*	14	38	4	62	101	20	239
*Ta*	18	39	6	75	176	21	335
*Tv*	16	42	4	74	162	20	318

Note.—A, aspartic proteases; C, cysteine proteases; G, glutamic proteases; M, metalloproteases, S, serine proteases; T, threonine proteases*.*

### Purifying Selection on Nutrition-Related Genes

**Fig. 5.—:**
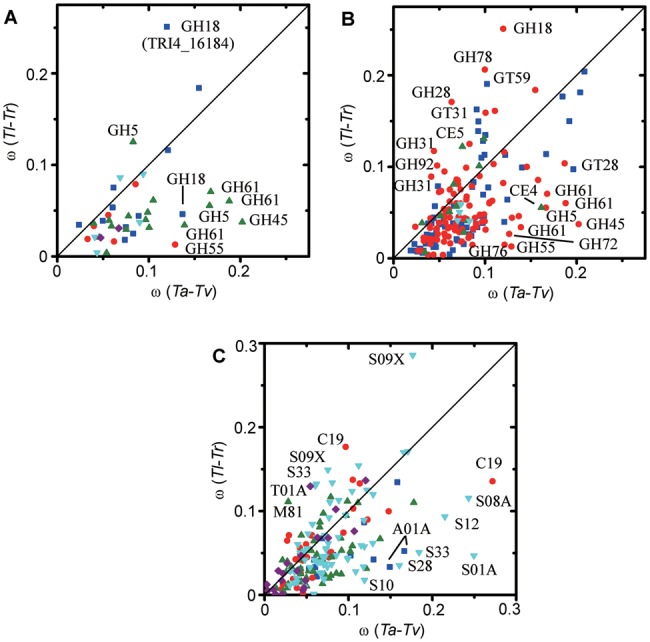
Comparison of selection pressure in *Tl–Tr* and *Ta* − *Tv*. (*A*) ω values for GH18 chitinases (blue squares), β-1,3/1,6-glucanases (red circles), cellulolytic enzymes (green up triangles), hemicellulolytic enzymes (cyan down triangles), and GH75 chitosanases (purple diamonds). (*B*) ω values for GH (red circles), GT (blue squares), CE (green up triangles), and PL (cyan down triangles). (*C*) ω values for aspartic proteases (blue squares), cysteine proteases (red circles), metalloproteases (green up triangles), serine proteases (cyan down triangles), and threonine proteases (purple diamonds). One data point for metalloproteases with ω(*Ta* − *Tv*) 0.107 and ω(*Tl–Tr*) 0.347 was omitted for clarity. Lower ω values indicate stronger selection pressure.

*Tl* is an efficient producer of cellulases and also a (potential) opportunistic human pathogen. Compared with the ancient nutrition strategy of mycotrophy, both the utilization of plant biomass and the utilization of nutrition from human are recently developed nutrition strategies. The similarity of *Tl* and *Tr* genome suggests that, the development of nutrition strategy of utilization of plant biomass seems to occur in their common ancestor. Furthermore, the above analyses suggest that the gene numbers for utilization of plant biomass are decreased in the common ancestor of *Tl* and *Tr*. Therefore, these genes are probably under stronger selection pressure in *Tl* and *Tr* than in *Ta* and *Tv*. The ratio (ω) of nonsynonymous substitutions per nonsynonymous site (d*N*) to synonymous substitutions per synonymous site (d*S*) can be used as an indicator of positive selection (ω > 1) and purifying selection (ω < 1). For each homologous group of nutrition-related genes from four species, we compared the ω values for the pair *Ta*–*Tv* and the pair *Tl*–*Tr* to check whether there is difference in the selection pressure. We calculated ω values for the nutrition-related enzymes, including chitinases, glucanases, cellulolytic enzymes, and hemicellulolytic enzymes (supplementary table S16, Supplementary Material online) and found that, all these enzymes have ω values much lower than 1 in both *Tl*–*Tr* and *Ta*–*Tv* (fig. 5*A*). Therefore, these genes are under purifying selection pressure rather than positive selection pressure.

Cellulolytic enzymes have a mean ω value of 0.103 (standard deviation 0.051, median 0.096) in *Ta*–*Tv* and have a mean ω value of 0.045 (standard deviation 0.026, median 0.039) in *Tl*–*Tr*. Among the 17 cellulolytic enzymes present in all the four species, 16 have a lower ω value in *Tl*–*Tr* than in *Ta*–*Tv* (fig. 5*B*). Statistical analysis indicated that the ω values of cellulolytic enzymes in *Tl*–*Tr* are significantly lower than that in *Ta*–*Tv* (one sample *t* test; null hypothesis: ω(*Tl*–*Tr*) − ω(*Ta*–*Tv*) ≥ 0; alternative hypothesis: ω(*Tl*–*Tr*) − ω(*Ta*–*Tv*) < 0; degree of freedom = 16; *P* = 0.0001). Therefore, consistent with above expectation, cellulolytic enzymes are subject to stronger purifying selection pressure in *Tl*–*Tr* than in *Ta*–*Tv*.

The ω values of hemicellulolytic enzymes in *Tl*–*Tr* are smaller than that in *Ta*–*Tv* but without statistical significance (one sample t test, null hypothesis: ω(*Tl*–*Tr*) − ω(*Ta*–*Tv*) ≥ 0; alternative hypothesis: ω(*Tl*–*Tr*) − ω(*Ta*–*Tv*) < 0; degree of freedom = 4; *P* = 0.096). For chitinases, the ω values in *Tl*–*Tr* are not statistically different from that in *Ta*–*Tv* (one sample t test, null hypothesis: ω(*Tl*–*Tr*) − ω(*Ta*–*Tv*) ≥ 0; alternative hypothesis: ω(*Tl*–*Tr*) − ω(*Ta*–*Tv*) < 0; degree of freedom = 11; *P* = 0.265). The comparison of ω values of mycoparasitism-related β-1,3/1,6-glucanases revealed that, ω values of these enzymes in *Tl*–*Tr* are smaller than that in *Ta* − *Tv*, though without statistical significance (one sample t test, null hypothesis: ω(*Tl*–*Tr*) − ω(*Ta* − *Tv*) ≥ 0; alternative hypothesis: ω(*Tl*–*Tr*) − ω(*Ta* − *Tv*) < 0; degree of freedom = 5; *P* = 0.059), indicating that these mycoparasitism-related β-1,3/1,6-glucanases probably play more important roles in *Tl*–*Tr* than expected.

We also calculated ω values for all the CAZymes (fig. 5*B*). It was shown that, most enzymes have smaller ω values in *Tl*–*Tr* than in mycoparasitic species, suggesting that the selection pressure on CAZymes are increased in *Tl*–*Tr*. Most enzymes have ω values ≤ 0.1 in *Ta* − *Tv*. A small number of enzymes have relative high ω values (> 0.1) in *Ta* − *Tv,* and their ω values are dramatically decreased in *Tl*–*Tr* (smaller than half of that in *Ta* − *Tv*, fig. 5*B*). It is also noted that, only about half of these enzymes belong to one of the above four classes of nutrition-related enzymes, suggesting that some other CAZymes are also important for the metabolism (probably nutrition-related) of *Tl*–*Tr*. Besides enzymes with decreased ω values, analyses also revealed enzymes with increased ω values in *Tl*–*Tr* (fig. 5*B*), suggesting lower selection pressure on these enzymes in *Tl*–*Tr*.

We also analyzed the selection pressure on proteases (fig. 5*C*). One sample *t*-test showed that, aspartic proteases (degree of freedom = 13; *P* = 0.0003), serine proteases (degree of freedom = 65; *P* = 0.0004), and metalloproteases (degree of freedom = 50; *P* = 0.0294) have decreased ω values in *Tl*–*Tr* than in *Ta* − *Tv*, suggesting that these proteases are under stronger purifying selection pressure in *Tl*–*Tr* than *Ta* − *Tv*.

## Discussion

Saprotrophic species *Tr* is a model for the study of *Trichoderma* physiology. Comparative genomics showed that, *Tr* has a smaller genome than the mycoparasitic species *Tv* and *Ta*, suggesting that gene loss events have occurred in the ancestor of *Tr*. In this study, we sequenced the genome of *Tl*, a close relative of *Tr*. Homology analyses and phylogenetic analyses suggest that the gene loss events occurred in the common ancestor of *Tl* and *Tr*. In addition, it is noted that, *Tl* has a smaller number of mycoparasitism-related genes, including CAZymes and NRPS/PKS, than *Tr*, suggesting that additional gene loss events occurred in *Tl* after the divergence from *Tr*.

The decrease of mycoparasitic ability can be affiliated to the decrease in the number of mycoparasitism-related genes. However, the development of the ability of saprotrophy on plant biomass seems not a result of acquiring additional genes for enzymes degrading plant biomass. *Tr* is an efficient producer of cellulases and hemicellulases and is used as the major industrial resource of these enzymes. *Tl* is also an efficient cellulase producer. However, comparison of cellulolytic enzymes and hemicellulolytic enzymes indicates that the number of these genes did not expand but was decreased in *Tl–Tr*. The ability of saprotrophy on plant biomass and the high efficiency of cellulolytic enzymes and hemicellulolytic enzymes production suggest that, these enzymes may have been optimized to improve the specific activities and/or expression levels in *Tl–Tr*.

Previous study has shown that several *Trichoderma* chitinase genes have codons under positive selection (Ihrmark et al. 2010). We calculated the ω values for the homologous groups and found that, all the analyzed chitinases, β-1,3/1,6-glucanases, cellulolytic enzymes, and hemicellulolytic enzymes have ω values smaller than 1, suggesting that these enzymes are under purifying selection pressure. Therefore, at the whole gene level, purifying selection dominates the evolution. Comparison of ω values shows that the cellulolytic enzymes have lower ω values in *Tl–Tr* than in *Ta* − *Tv*. In contrast, ω values of chitinases in *Tl–Tr* are not statistically different from that in *Ta* − *Tv*. The above results indicate that, the nutrition strategy of saprotrophy on plant biomass imposes a strong selection pressure on cellulolytic enzymes.

## Conclusions

*Tl* has a genome of 31.7 Mb, smallest among the four sequenced species. *Tl* has the closest relationship with *Tr* among the sequenced species. Gene loss events probably occurred in the common ancestor of *Tl* and *Tr*, resulting in the smaller genome size of *Tl* and *Tr* than that of *Tv* and *Ta*. Development of new nutrition style is not only related to the decrease of nutrition-related genes (especially for fungal cell wall polysaccharides-degrading enzymes) but also related to the increase of selection pressure on nutrition-related genes (especially for plant cell wall polysaccharides-degrading enzymes). This study provides insights into the physiology and evolution of nutrition strategy of *Trichoderma* and is helpful for development of improved biocontrol strains and cellulases and hemicellulases-production strains.

## Supplementary Material

Supplementary tables S1–S17 and figure S1 are available at *Genome Biology and Evolution* online (http://www.gbe.oxfordjournals.org/).

Supplementary Data
