# Supplementary material for: Comparative Genomics Provide Insights into Evolution of Trichoderma Nutrition Style
Source: Genome Biol Evol. 2014 Jan 29;6(2):379–90. doi: 10.1093/gbe/evu018 (PMC3942035; doi:10.1093/gbe/evu018)
Supplement: Supplementary Data [file supp_6_2_379__index.html]

Comparative genomics provide insights into evolution of Trichoderma nutrition style — Comparative Genomics Provide Insights into Evolution of Trichoderma Nutrition Style — Supplementary Data 

# Comparative Genomics Provide Insights into Evolution of *Trichoderma* Nutrition Style

## Supplementary Data

files

**Files in this Data Supplement:**

- Supplementary Data - xls file
- Supplementary Data - doc file
