# Supplementary material for: Comparative Genomics Provide Insights into Evolution of Trichoderma Nutrition Style
Source: Genome Biol Evol. 2014 Jan 29;6(2):379–90. doi: 10.1093/gbe/evu018 (PMC3942035; doi:10.1093/gbe/evu018)
Supplement: Supplementary Data [file supp_evu018_suppl_gbe_2-rev-rev.doc]

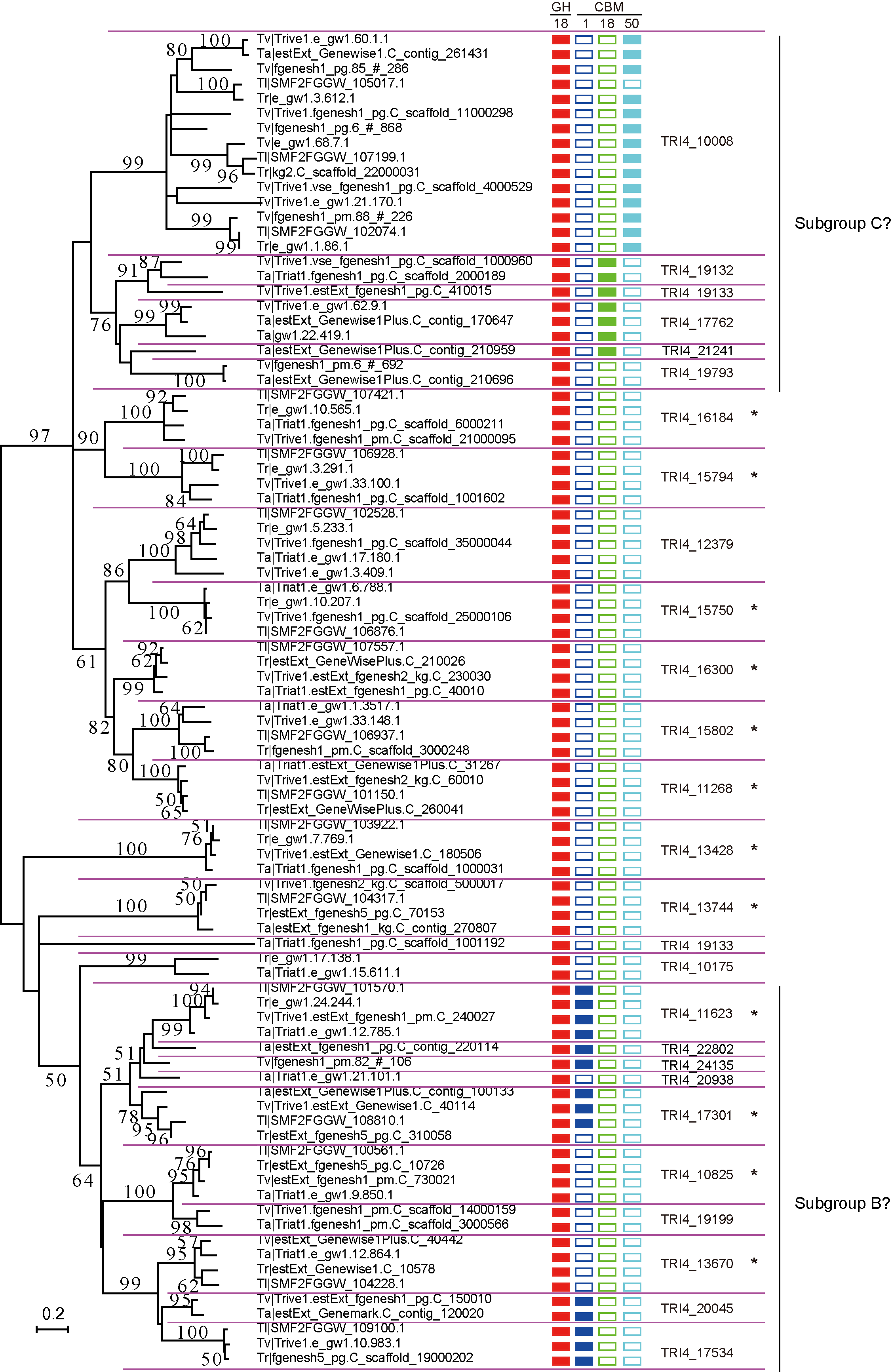


Figure S1. A consensus Neighbor-Joining tree of GH18 chitinases annotated in *Trichoderma* genomes. Left panel, a consensus neighbor-joining tree constructed based on 106 sites using JTT matrix and 500 bootstrap replicates. The bar represents 0.2 substitutions per site. Bootstrap percentages (≥ 50%) were shown on the branches. Species abbreviations and sequence names are shown on the right of the tree. Species abbreviations: *Ta*, *Trichoderma atroviride*; *Tl*, *Trichoderma longibrachiatum*; *Tr*, *Trichoderma reesei*; *Tv*, *Trichoderma virens*. Middle panel, Pfam domains annotated using Pfam database. Presence of a domain is indicated using a filled box and absence using an open box. GH18, Glyco_hydro_18; CBM1, CBM_1; CBM18, Chitin_bind_1; CBM50, LysM. Right panel, homology group IDs based on OrthoMCL analysis and subgroups based on Pfam domain structures. The homology groups with one sequence from each species are indicated using asterisks. Three sequences were not included in the tree because of short sequence length or long distance from other sequences.
